# Some Therapeutic Uses of Water*Read before the Brazos Valley Medical Association, on November 13, 1901, at Bryan, Texas.

**Published:** 1902-07

**Authors:** L. M. Bassett

**Affiliations:** Hearne, Texas


For Texas Medical Journal.

### Some Therapeutic Uses of Water.*

BY L. M. BASSETT, M. D., HEARNE, TEXAS.

In an able article in the *Journal of the American Medical Association,* September 21st, Dr. Fenton B. Turck, of Chicago, urges upon the medical profession of the United States the absolute necessity of a more thorough knowledge of the therapeutic value of dietetics hydrotherapy and physico mechanical therapeutics, and

---

*Read before the Brazos Valley Medical Association, on November 13, 1901, at Bryan, Texas.

the desirability of rescuing these valuable agents from the hands of the quacks and the establishment of the teaching of their principles in the regular college curriculum.

Current works on therapeutics usually give but scant space to these subjects.

While not willing to under-value the importance of massage, hygienic gymnastics and practical dietetics, to my mind hydrotherapy intelligently applied is a powerful therapeutic agent, of whose value we of the rank and file of the medical profession avail ourselves entirely too seldom.

Dr. Turck says: "The very important subject of hydrotherapy which comprises the application of water internally and externally from solid to fluid and vapor, from ice to steam, depending for the result upon the judicious application of its mechanical and thermal influences, is being entirely too much neglected."

Baruch, in an admirable article in the *Therapeutic Gazette* for September, after calling attention to the fact that water and regimen were the only two remedial agents of the fathers of medicine which had survived the searching investigation of modern physiologic criticism, remarks, in explanation, "That the action of water alone is based upon physiological principles; possesses a physiologic basis which becomes more and more apparent as our insight into the nature and course of diseased processes is made more and more clear by modern investigation."

Continuing, he explains, "We know today that hydrotherapy is but the application of temperatures through the medium of water which, by reason of its physical property, absorbs or gives off temperatures very readily. Cold and heat are irritants, which act as physiological stimulants when mild, and which are depressants to the point of tissue destruction when intense."

Bearing these facts in mind, and by judiciously arousing these well known effects in the sensory terminals of the vascular area of the skin, peripheral stimulation is evoked which, like all such impressions, is conveyed to the central nervous system and reflected upon the life-sustaining organs.

Briefly stated, the principle of hydrotherapy consists in the evoking of effects upon the internal life-sustaining organs from effects produced by the application of water to the skin.

These effects, according to Kellog, are definite and varied, and may be calculated upon as positively as the sounds elicited by a musician from the keys of an instrument.

The great latitude afforded by water for the application of temperatures from the brief sprinkling of the face in syncope to the prolonged immersion in typhoid indicates a latitude in dosage available in no other remedy.

In his recently published work, Kellog enunciates the principles of hydrotherapy which, epitomized, are as follows:

Water possesses no specific curative virtue.

Its use is simply as a convenient vehicle by which thermic impressions may be made, either by communicating heat to the body or abstracting heat from it.

It is more universally applicable than any other therapeutic agent, and is capable of producing a greater variety of effects than any other therapeutic agent known to man.

Powerful and instantaneous impressions can, with certainty, be made upon distant organs by suitable hydriatic application made to the surface of the skin. Functional activity may be increased, diminished and controlled at will.

The familiar expedient of bathing the face, head and eyes to relieve the effects of heat and fatigue, or overcome drowsiness, does not depend upon the local effect of the water upon the skin, but the circulation of the brain itself is affected and the supply of blood is lessened, the venous stasis is relieved, the poison from accumulated toxines eliminated and thus the mind irrigated and refreshed.

The dashing of cold water into the face of the fainting woman in like manner brings back the color to the palid cheeks. Thus we see how the cooling of the myriad nerve-endings in the skin sends to the brain a storm of sensory impressions which react upon the centers of the brain and instantly result in the bringing back of the patient to consciousness.

A dash of cold water upon the chest will in like manner restore suspended respiration.

Cold water to the abdomen or feet will arouse a desire to urinate. Cold water in like manner over the bowels, liver or stomach will stimulate these organs to functional activity.

It is thus readily seen that the effects of cold water are not, as some have assumed, only to reduce temperatures in fever, but that it has a much wider application, and is in reality, as stated by Turck, a physiological stimulant of the greatest importance and the *antipyretic effect is in reality secondary to its nerve stimulating effect.*

Whoever, he asks, has seen the dull eyes of the typhoid fever patient brighten after the cold friction bath; whoever has seen the dicratic galloping pulse gain in force and diminish in frequency; whoever has noticed the deadly pallor of the trunk and the cyanotic face disappear; whoever has observed the enormous increase in amount and toxicity of the urine after a properly administered cold bath, must be convinced that he stands in the presence of a powerful and salutary agent the like of which does not exist elsewhere in medicine. And Baruch likens the revivifying effects of the cold friction bath in typhoid to the "effect of a cooling shower to parched and thirsty vegetation."

In the hydriatic management of fevers, he adopts the following scheme of *dont's* as a summary of what to do and what not to do:

First. Don't bathe in cold water to reduce fever, but to refresh the fever-stricken patient.

Second. Don't permit cyanosis or chattering of teeth. Stop!

Third. Don't stop bathing because patient complains of chilliness unless teeth chatter.

Fourth. Don't raise temperature of bath on account of chilliness. Shorten bath and increase friction.

Fifth. Don't neglect friction during every cold procedure; it prevents chilling.

Sixth. Don't disregard the fact that the Brand bath at 65° to 70° is the ideal bath for typhoid.

Seventh. Don't use the Brand bath in a bath room.

Eighth. Don't give up bathing because the ideal bath is not procurable. Other procedures are useful.

Ninth. Don't use ice coil to abdomen. It has no refreshing effect and renders skin cyanotic beneath it.

Tenth. Don't lose sight of the fact that the chief aim of all cold procedures is reaction.

These directions may also be found useful in many other conditions than fever.

In all cases it is to be remembered that the cold bath properly applied should have a distinctly tonic and invigorating effect directly and indirectly to the nervous, muscular, circulatory and digestive systems, resulting in increased hematosis, better nutrition of the skin and glandular apparatus and their consequent increased functional activity and eliminating capacity, and allow me to say here, that fever is caused not so much by the poisons generated in the system, but by inability to throw it off. The increase of functional activity after the cold procedure extends not only to the skin, but to the liver and to all the internal organs and glands of the body.

The prompt and positive action of cold water as a cardiac and respiratory stimulant should insure resort to it at once in sunstroke and all emergencies attended with syncope and sudden or violent interruption of respiration and circulation.

For all acute infectious diseases the cold bath is indicated. Brand immerses the patient at once in a bath of the temperature desired, while Von Ziemesen begins with a temperature of 90° and gradually lowers the heat while the patient is in the bath.

Under all circumstances, no matter what method of applying cold water, active friction of the surface should be kept up while the patient is in the bath to stimulate the circulation and prevent chilling.

As before stated, no attention need be paid to the complaints of the patients unless cyanosis or chattering of the teeth occur, in which case the bath should be immediately stopped, the patient warmly wrapped in bed with hot bottles to the feet and a stimulant internally or hypodermically.

When giving the bath the room should be moderately warm and free from draughts.

The immediate effects of the cold bath upon the temperature is a slight rise, due to the sudden driving of the blood to the internal organs by the effect of the cold on the skin. This is followed in a short while by a decided fall, caused by the active dilatation of the cutaneous capillaries following their early contraction.

The effect of the bath on the nervous system is manifested in the lessened delirium and prostration and the quiet sleep which usually follows.

Since the introduction of the hydriatic treatment of typhoid, the mortality has been reduced from twenty-five per cent. under the expectant plan to three and five-tenths per cent. as reported by Brand himself, and others have reported even more brilliant results.

To secure such results treatment must be begun early in the disease, though the baths are valuable at any stage unless prostration from severe hemorrhage or collapse from perforation are present.

In administering the cold bath for no matter what disease, it is important that the body should not, under any circumstances, be allowed to become chilled before entering the bath. Such a condition favors cramps and tetanic contractions of voluntary muscles, and satisfactory reaction cannot be secured.

In fine, Foster cautions us to remember that any agent which is capable of so profoundly impressing the system for good must be equally potent for evil, and advises that the effects of the bath be cautiously watched and a temperature and mode of procedure selected that will be devoid of danger.

In labor and broncho-pneumonia, Baruch recommends a bath at 95°, reduced gradually to 80°, with friction, and claims to be able to abort the disease and hasten resolution. The bath is continued for fifteen minutes, and is given preference over every other form of treatment in those cases especially where the heart is weak. Reduction of temperature, restoration of heart's action and deepened respiration and restoration of nervous tone and capillary circulation are the effects obtained. It is well to always give a heart stimulant in pneumonia before entering the bath.

In scarlet fever, there is much testimony in favor of the use of the bath. There is no claim that complications are avoided, but that the appetite and mental condition in severe cases are much benefited.

Diphtheria has been treated by the bath with good results, many observers testifying to the value of warm baths with local cold applications in increasing depth of respiration and favoring vigorous coughing. The use of the antitoxin treatment has, however, practically superceded all other treatment in this disease.

Bathing with water at 70° to 80° with cold to the head is of great value in cholera infantum, more particularly in those cases complicated with congestion of the brain.

Foster, in cerebro-spinal meningitis, pronounces the effect of cold water excellent for relieving restlessness and producing sleep, but on account of the evil effects of disturbing the patient by moving into tub, advises resort to the cold sponge or wet pack in preference. The object obtained is reduced fever, lessened restlessness and increased tone of the nervous system.

In all cases of sepsis with high fever from no matter what cause, the use of cold water is indicated.

Liebermeister recommends it in smallpox, acute articular rheumatism, puerperal sepsis, quinsy, erysipelas and in all cases with febrile symptoms of long standing.

In the early stages of tuberculosis, combined with exercise, fresh air and wholesome diet and regular habits of rest, much has lately been accomplished. In chronic organic nervous diseases, as tabes dorsalis, myelitis, epilepsy and paraplegia, cold water acts favorably.

In extensive burns, cold water is used by Hebra in the Vienna General Hospital.

Fraser recommends the continuous immersion of the part in water in the treatment of infected wounds, cellulitis and gangrene, claiming that it will almost instantly limit gangrene and control resulting septicemia and spanemia. It also quickly relieves cellulitis and phlegmonous inflammations, reducing temperature and pulse and overcoming depression of the vital forces. In such cases he advises the prolonged submersion of the part in water below the temperature of the room, claiming that many bacteria will not develop at all at a temperature slightly below the temperature of the room, and others which do develop will, in a great measure, be deprived of their virulence, which would thrive vigorously at a slightly higher temperature.

According to Kellog, cold water is the best known means of stimulating the blood-making organs of the body. Its action is much the same as cold air, but has the great advantage that it is capable of better regulation and dosage, and is available at all times and places. It is consequently of greatest value in anemia.

In chronic malarial diseases, he rates it above all other remedies in securing permanent results. Its suitable employment alone, he claims, will often interrupt the paroxysm, and when used in connection with quinine, the effective dose of the latter may be reduced one-half, and in the course of a few days dispensed with altogether.

In diabetes, cold water stimulates oxidation and burns up sugar.

In obesity hot baths followed by cold douches and exercise increases the oxidation of fats.

The Charcot douche, which is simply the cold daily spinal douche, has worked wonders in the treatment of neurasthenia.

There is, in fact, says Kellog, scarcely a malady known in which water may not render incalculable service. Cold applications stimulate every cell and fiber of the body; hot applications relax; neutral applications relieve irritation and produce powerful sedative effects.

As this paper has drawn itself out already to undue length, I will not undertake to review the effects of water internally, nor by vaginal or rectal douche, nor by hypodermoclysis of normal saline solution, but will bring it to a close by describing in as few words as possible the manner of administering a few cold water procedures.

By the cold bath in this paper is meant a tub bath with water at a temperature of 65° to 70°, though occasion may occur where a much lower temperature will be required. The tub should be brought near the bed and all draughts of air excluded and the room well warmed. If patient is perspiring he should be thoroughly dried. The body should be immersed in the cold water up to the chin and cold compresses kept to the head, which should be supported upon a rubber pillar or other device. In case of a child, the head may be supported by the nurse. The body should be immersed quickly to lessen shock. An ounce of whiskey should be given before the bath. Care should be had that the chest and shoulders are completely covered with water to guard against pulmonary congestion. During the bath vigorous rubbing of the skin of both trunk and extremities is kept up, except that in typhoid and dysentery massage of the abdomen should not be undertaken. The duration of the bath should be from five to twenty minutes for an adult and usually from five to ten minutes for a child.

Cyanosis of the face or chattering of the teeth should not be permitted, and should it occur the bath must be immediately terminated and hot applications made as hereafter directed, a stimulant given and the patient transferred to bed and warmly wrapped with a blanket. When reaction has been established the patient should be thoroughly dried with rough towels and comfortably covered with the bed clothes. One hour after the bath the temperature should be taken and the bath repeated whenever the temperature has again reached 102-5°.

Great gentleness should always be used, and the patient should be submerged at once in the bath to terminate the shock and feeling of fear associated with it. All fuss should be prohibited.

While the tub bath is the orthodox way of administering the Brand bath, it is seldom convenient in a country practice, while the cold wet sheet and the dripping sheet are practically always available and the method of application simple. I shall, therefore, give a brief description of their technique as recommended by Kellog.

*Requisites.*—A linen sheet, a Turkish sheet, two towels, a tub containing hot water for the feet, a pail of water at 70° (water at a higher or lower temperature may be employed if indicated).

The patient being stripped (the head is cooled in the usual way and protected by a cold wet cloth) with a dry sheet thrown around him, the patient steps in a tub containing hot water for the feet. The attendants, two in number, prepare the wet sheet, which is wrung dry enough not to dry too rapidly. When this sheet is ready one of the assistants, holding one end of the sheet properly gathered in his right hand, and seizing the upper left hand corner with his left hand, steps in front of the patient, while the other attendant steps behind him and withdraws the dry sheet and stands ready to assist.

The patient raises both arms, while the attendant in front places the upper left hand corner of the sheet under his right arm. The patient, lowering the arm, holds the sheet in place. The attendant passes the sheet across the chest and front of the body beneath the left arm, which is then lowered also. The sheet is now carried around the body with the assistance of the attendant, who stands behind the patient and pulls the bottom around. As the sheet is brought across the back of the patient the attendant reaches over and seizes the sheet just above the point of the right shoulder and pulls it first upward then downward upon the patient's chest while with the other hand he carries the sheet across the chest, covering the fold, and over the left shoulder deftly tucking the corner under the edge of the sheet behind. The attendant behind tucks the sheet between the patient's legs, which are then brought closely together. The sheet is thus brought everywhere in close contact with the skin.

As soon as the patient is completely enveloped, an operation requiring from five to eight seconds, both attendants begin to rub vigorously, covering the whole surface as quickly as possible—one the legs and hips, the other the trunk and arms. The rubbing should be continued from one to three minutes, or until the sheet is everywhere thoroughly warmed. The attendants should bear in mind that the patient is not to be rubbed *with the sheet,* but over the sheet with a downward percussion stroke.

The bath does not depend for its effect upon the irritation of the skin by friction of the sheet, but upon *thermic stimulation* and the assistance to the cutaneous circulation afforded by the intermittent pressure upon the surface vessels.

The rubbing movements are made by a sort of glancing percussion stroke from above downward.

Percussion may be applied over the fleshy parts and gentle patting to parts that are sensitive to vigorous rubbing.

Care must be had that the whole surface of the body is gone over many times in rapid succession. The rubbing must be continued with vigor during the entire application, and stout attendants will, at the end of the procedure, find themselves quite out of breath if they have done their duty.

When the sheet is well warmed, it is dropped and the Turkish sheet is thrown around the patient and the bath is completed by vigorous rubbing over the Turkish sheet followed by dry friction with the hand with or without oil rubbing.

The intensity of this bath may be varied in a number of different ways: (1) By the temperature of the water employed. (2) By the amount of water left in the sheet. (3) By using a thinner or a thicker sheet. (4) By employing two or more wet sheets in succession, changing as soon as warm. (5) By the vigor of the friction movements applied, and (6) by the duration.

This fact renders the rubbing wet sheet a most convenient measure for use outside a regular hydriatic establishment, or where convenient appliances for treatment are not at hand.

The longer the continuation of the bath the greater will be the thermic effect produced, hence when excitement of the nerve centers and tissue change is undesirable, the sheet should be wrung dry, the temperature of the bath should not be too low, the duration should be short and the rubbing vigorous.

For feeble or bed-ridden patients, the rubbing wet sheet may be applied in bed. Care should be had to apply hot bags or bottles to the feet during the procedure.

For bed-ridden patients, the sheet may be applied in much the same way as when the patient stands.

The wet sheet being spread upon the bed over a blanket, the patient is placed upon it. He raises his arms over his head while one side of the sheet is passed across the body, the edge being tucked under the trunk and between the legs. The arms are then lowered and the other half of the sheet is drawn across the body so as to include both arms and to cover the shoulders, the free corner being tucked well under the shoulder, care being taken to exclude the air. The patient is then vigorously rubbed from head to foot, the whole surface being gone over as rapidly as possible. Should there be at first a tendency to chill from too rapid evaporation, a warm blanket should be drawn over the wet sheet and the friction applied beneath it. The rubbing should be continued until the surface of the body is well reddened and the sheet thoroughly warmed, usually one to three minutes. The sheet should then be removed and the patient covered at onc with a linen or Turkish sheet and blanket and dried beneath the blanket.

In cases of visceral congestion or cerebral hyperemia, in order to avoid retrostosis the patient should stand in very warm water, 104° to 109°, during the procedure. The rubbing wet sheet may be applied several times a day to secure desired results. Before applying the cold rubbing sheet care must be taken to see that the patient's skin is in the proper condition to receive a cold application with benefits.

If the skin is cold it must be warmed by the hot shower bath or by some other heating process. The wet sheet rub should never be given if the patient's skin is cold.

The dripping sheet is a much more vigorous thermic application than the rubbing wet sheet. For its application, two or three pailfuls of water at different temperatures should be had in readiness. The sheet is applied thoroughly saturated with water and dripping. The patient is not rubbed, but vigorously spatted for twenty or thirty seconds, then half a pailful of water at a little lower temperature than that of the sheet is poured over each shoulder and the percussion then resumed. When evidence of reaction appears in returning warmth another pailful at a still lower temperature may be poured over the patient if desired, but so vigorous an application is seldom indicated.

The usual temperatures employed are 70° for the sheet, 65° for the first pail and 60° for the second pail.

If higher temperatures are employed, it is difficult to get good reaction, since the exciting effect of the cold is necessary to arouse the nerve centers to the degree required to produce the thermic and circulatory reaction which this procedure is intended to provoke.

Energetic percussion should be maintained outside the sheet to keep up the cutaneous circulation. The bath may be prolonged until the desired effect is obtained.

Care must be taken to stop the bath if the patient begins to lose the sensation of warmth which follows the first chill from the contact with the wet sheet, or is threatened with blueness of the skin or chattering of the teeth with shivering.

For cases unable to stand up, or where for any cause it is considered unwise to permit it, place first upon the bed a rubber sheet, upon which spread a linen sheet wet with water at 75° and wrung lightly.

Place patient on the sheet and envelop him quickly as for a wet sheet pack; then let two attendants proceed to encourage circulatory reaction by gentle percussion and gentle rubbing over the whole surface, giving special attention to the extremities. As the sheet is warmed, cool by splashing water at 60° or 70°, continuing the rubbing and splashing until patient begins to shiver. Then remove wet sheet, wrap in dry linen or Turkish sheet, cover with a woolen blanket and after fifteen minutes take the rectal temperature. Renew bath when temperature reaches 102-5° F.

This procedure slows the pulse and at the same time energizes the heart, and, contrary to the usual physiologic law, while the pulse is lowered respiration is increased both in depth and frequency.

Thermic reaction is usually less marked than in the immersion bath, circulatory reaction predominating, but thermic effects are obtainable at will in any degree of intensity.

The very pronounced and lasting circulatory reaction produced secures a vigorous cutaneous circulation and an increased rate of blood movement throughout the whole body.

The effect of this procedure is intermediate between the douche and the wet sheet pack.

At the moment of application gasping respiration is produced by this procedure, but these sensations give place to prolonged and deep respiratory movements with slowing of the heart and a general feeling of warmth and glowing of the skin as reaction begins.

The combination of the thermic effects of cold with the mechanical effects of friction produces at first a general contraction of the small cutaneous vessels, quickly followed by dilatation.

The first period of contraction of the vessels of the skin coincident with the dilatation of the vessels of the brain, lungs and abdominal viscera is quickly followed by dilatation when the blood returns to the skin with the reaction. Blood pressure is increased and also muscular capacity, assimilation of nitrogen is increased, appetite improved and temperature temporarily lowered.

Kellog assures us that by the measures outlined above we shall be enabled to obtain all the good effects of the Brand bath, and they have the decided advantage that no apparatus is necessary, and they may be employed under almost any surroundings.

The important point to be remembered about all of these procedures is the necessity for continued and vigorous friction, which maintains the circulation of the blood in the skin and prevents chilling or internal congestions. If this point be remembered, then the use of colder or warmer water, dryer or wetter, or single or double sheets, together with the shortening or the prolongation, the process will easily enable us even in the country to realize many of the good effects of hydriatic treatment to which we have heretofore been almost strangers.

The contraindications to cold hydriatic procedures are extreme hyperesthesia of the skin. General neuritis and severe neuralgias involving large nerve trunks and extreme feebleness of the circulation, such that proper reaction cannot be secured, and even in many of these cases milder measures may be employed until reaction is improved.

